# Shared Humanity: Advancing the Personhood of People Living With Dementia

**DOI:** 10.1093/geroni/igab046.1536

**Published:** 2021-12-17

**Authors:** Steven Sabat

**Affiliations:** Georgetown University, Georgetown University, District of Columbia, United States

## Abstract

During the past three decades, the idea of personhood, and the degree to which people living with dementia (PLWD) possess it and ought to be treated as such, has been discussed by a number of important scholars such as Tom Kitwood and John Swinton. Although both asserted that PLWD ought to be treated as persons, their notions of personhood appear to be quite different. Kitwood noted that personhood was a status “bestowed” on another individual, whereas Swinton asserted that personhood was endemic to human beings. Yet, these approaches are complementary. I show, using case studies, how supportive communities are required for PLWD to teach us about our humanity, so long as we remain “finely aware and richly responsible” (Nussbaum, 1990). Our humanity is shared, in the Zulu concept of “Ubuntu” (Tutu, 1981;1989), when each individual’s humanity is ideally expressed in relationship with others. This has important implications for clinical practice.

